# Alcohol, Neural Stem Cells, and Adult Neurogenesis

**Published:** 2003

**Authors:** Fulton T. Crews, Kim Nixon

**Affiliations:** Fulton T. Crews, Ph.D., is director of the Bowles Center for Alcohol Studies and a professor in the Departments of Pharmacology and Psychiatry at the University of North Carolina, Chapel Hill. Kim Nixon, Ph.D., is a postdoctoral fellow at the Bowles Center for Alcohol Studies at the University of North Carolina, Chapel Hill

**Keywords:** neural cell, stem cell, cell growth and differentiation, neurobiological theory of AODU (alcohol and other drug use), genetic theory of AODU, biological regulation, environmental factors, stress, neurochemistry, glutamate receptors, serotonin receptors, limbic system, hippocampal formation, chronic AODE (alcohol and other drug effects), brain, morphology, adult

## Abstract

Recent research demonstrates that neural stem cells divide throughout life and give rise to new neurons, a process known as neurogenesis. This article addresses two principal questions concerning alcohol and adult neurogenesis: To what extent are neurogenesis in the adult brain and the risk for alcoholism governed by similar factors? And, to what extent and through what mechanisms do alcohol use and alcoholism affect adult neurogenesis? This article also discusses genetic and environmental influences on risk for alcoholism and on regulation of neurogenesis; the possibility that modulation of neurogenesis contributes to alcoholic pathology; and the evidence that alcohol disrupts neurogenesis in the adult brain, and the neurochemical processes by which this may occur.

For decades, the majority of neuroscientists believed, and physicians were taught, that the number of nerve cells (i.e., neurons) in the adult brain was fixed early in life and that learning and other flexible (i.e., plastic) processes in the brain must be related to changes in the existing neurons. Hebb (1949) postulated that nervous system plasticity was achieved by “strengthening synapses” without adding new neurons. The theoretical understanding of plastic processes such as learning, memory, mood, and other features of adult behavior is entrenched in this concept of a fixed number of neurons in the adult brain. As a result, research on brain plasticity has long focused on alterations in neurotransmitter receptors, numbers of synapses, structure of synapses, and transmitter release mechanisms.^1^

The seminal discoveries on the formation of new neurons (i.e., neurogenesis) in adulthood were made in the 1960s (Altman and Das 1965). Until recently, dogma and insufficient technology prevented acceptance of these findings as an additional process influencing brain plasticity. This area remains controversial, with researchers currently debating how extensive neurogenesis is in the adult brain (Rakic 2002). Recent research clearly establishes that neural stem cells (NSCs) divide throughout life and give rise to new neurons in at least two regions of the adult brain: (1) in the dentate gyrus of the hippocampus, a brain region important for learning and memory, and (2) in the subventricular zone (SVZ) of the anterior lateral ventricles, the site of origin for olfactory bulb neurons. (See the sidebar “What Is a Stem Cell?” for more detailed information about these cells and, in particular, the role of NSCs in the central nervous system.) The function of adult NSCs is not known, but they are associated with complicated brain functions such as learning, mood, and association of sensory information. The discovery of NSCs and adult neurogenesis provides a new theoretical framework for understanding processes regulating brain plasticity (Gage 2000). As addiction is thought to represent maladaptive changes in brain plasticity, understanding the role of alcohol-induced changes in the brain and exploiting the new research findings on brain plasticity should be included in scientists’ schema for understanding, treating, and curing alcoholism.

**What is a Stem Cell?**

***Types of Stem Cells***

Stem cells are cells that can divide indefinitely, renew themselves, and give rise to a variety of cell types. There are several different types of stem cells. Totipotent stem cells (i.e., those present in the earliest stages of embryonic development) can produce all cells of an organism. Pluripotent stem cells (another type of embryonic stem cell) are capable of giving rise to every cell type except the trophoblasts of the placenta. Multipotent stem cells, including neural stem cells (NSCs), are more restricted in the types of cells they are capable of producing or becoming. A totipotent (embryonic) stem cell may first become a pluripotent stem cell and then eventually a multipotent stem cell through a series of events called fate restrictions, which limit the types of differentiated cell it can become.

***Neural Stem Cells***

A variety of adult neural cell populations have been identified as stem cells, although the exact characterization and definitions of these populations are evolving. It is generally accepted that NSCs self-renew and are potentially multipotent in that, under the right conditions, they can produce other cell types and give rise to several types of cells in the central nervous system, including neurons and other brain cells. In this report NSC is used in a general way to refer to adult brain cells that divide to form differentiated nervous system cells (Nowakowski and Hayes 2001).

The role of stem cells in central nervous system development is well described (Kennea and Mehmet 2002), but their existence in the adult brain was confirmed only recently (Gage 2000). In vitro studies have been able to culture NSCs from many regions of brain. Furthermore, brain insult studies have suggested that NSCs are present in all brain regions but normally are suppressed from dividing. Under normal conditions, however, continuous neurogenesis occurs primarily in two discrete brain regions, each of which contains NSCs. It is well accepted that NSCs underlie adult neurogenesis in the subventricular zone (SVZ) of the anterior lateral ventricles and the dentate gyrus of the hippocampus (see the figure in this sidebar).

**Figure:**
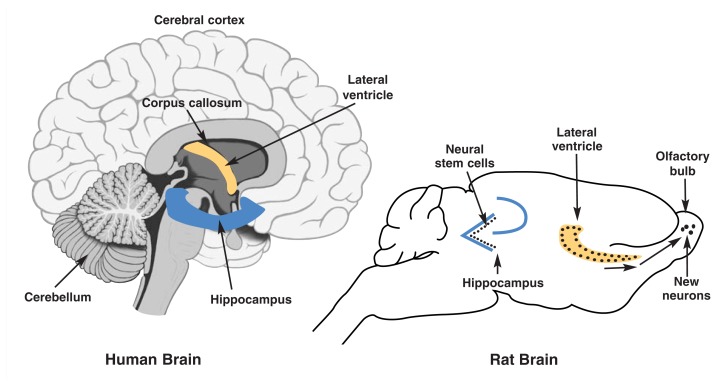
Sites of adult neurogenesis (rodent studies) compared with appropriate human brain regions. Neurogenesis has been confirmed in two regions of the adult brain: the subventricular zone (SVZ) of the anterior lateral ventricles (the site of origin for olfactory bulb neruons) and the dentate gyrus of the hippocampus (a brain region involved in learning and memory). In the SVZ, progenitor cells migrate to the olfactory bulb, where they differentiate into neurons. In the dentate gyrus, cells divide along the subgranular zone (also see the figure in the sidebar, p. 201) and migrate into the granule cell layer before terminally differentiating into granule cells.

Neurogenesis in the SVZ occurs when cells divide along the SVZ, then migrate to the olfactory bulb, which is involved in the sense of smell (Alvarez-Buylla and Temple 1998). Hippocampal NSCs also originate in the walls of the lateral ventricles during embryonic development but migrate out to begin forming the hippocampus. Granule cells within the dentate gyrus of the hippocampus do not begin forming until mid-gestation, peaking during the third trimester (in humans, or its equivalent in other organisms) (Bayer 1980). Granule cell neurogenesis continues through adulthood, declining with age in rats and in humans (Altman and Das 1965; Gage 2000). In the dentate gyrus of the hippocampus, hundreds of thousands of new granule cells—up to 6 percent of the total adult population—are formed each month, though equivalent cell death has not been observed with current techniques (Cameron and McKay 2001).^1^ New cells in the dentate gyrus divide along the subgranular zone during early development, migrate into the granule cell layer, and become neurons in shape (morphology) and gene expression (phenotype). These new cells express neuronal markers, show electrophysiological characteristics of neurons, and make appropriate connections to carry information to and from other parts of the brain (Gross 2000).

Several findings suggest that NSCs are important to adult brain plasticity. First, thousands of new brain cells are formed daily, and most of them are integrated as new functional neurons (Cameron and McKay 2001). Second, this process occurs across many species, including humans (Gould 1999). Third, environment, genetics, and drugs alter neurogenesis in a manner that is consistent with NSCs having an impact on plastic processes such as learning and mood (Duman et al. 2001; Gould 1999). Finally, newborn neurons alter brain structure and circuitry, a process historically thought to occur only in the developing brain or through growth of existing adult neurons.

—Fulton T. Crews and Kim Nixon

ReferencesAltmanJDasGDAutoradiographic and histological evidence of postnatal hippocampal neurogenesis in ratsJournal of Comparative Neurology12433193351965586171710.1002/cne.901240303Alvarez-BuyllaATempleSStem cells in the developing and adult nervous systemJournal of Neurobiology36210511019989712298BayerSADevelopment of the hippocampal region in the rat. I. Neurogenesis examined with ^3^H-thymidine autoradiographyJournal of Comparative Neurology190871141980738105610.1002/cne.901900107CameronHAMcKayRDAdult neurogenesis produces a large pool of new granule cells in the dentate gyrusJournal of Comparative Neurology435440641720011140682210.1002/cne.1040DumanRSMalbergJNakagawaSRegulation of adult neurogenesis by psychotropic drugs and stressJournal of Pharmacology and Experimental Therapeutics2992401407200111602648GageFHMammalian neural stem cellsScience28754571433143820001068878310.1126/science.287.5457.1433GouldESerotonin and hippocampal neurogenesisNeuropsychopharmacology21Suppl 246S51S19991043248810.1016/S0893-133X(99)00045-7GrossCGNeurogenesis in the adult brain: Death of a dogmaNature Reviews Neuroscience11677320001125277010.1038/35036235KenneaNLMehmetHNeural stem cellsJournal of Pathology197453655020021211586910.1002/path.1189NowakowskiRSHayesNLStem cells: The promises and pitfallsNeuropsychopharmacology25679980420011175017410.1016/S0893-133X(01)00379-7

1The dentate gyrus does not appear to expand sufficiently to accommodate all the new neurons formed throughout adulthood, suggesting some form of granule cell turnover in the adult hippocampus.

This article addresses two principal questions concerning the connection between alcohol and adult neurogenesis. First, to what extent are neurogenesis in the adult brain and the risk for alcoholism governed by similar genetic and environmental factors? Second, do alcohol use and alcoholism affect adult neurogenesis, and if so, what are the mechanisms underlying those effects?^2^

## Genetic and Environmental Regulation of Adult Neurogenesis and Alcoholism

The components of neurogenesis—the proliferation of NSCs and their survival and differentiation into neurons and other brain cells—are heavily regulated by genetics but also respond to environmental factors. Indeed, many of the environmental factors that regulate adult neurogenesis also are affected in people with chronic alcoholism. Thus, the regulation of NSCs is similar to some aspects of alcohol abuse and alcoholism. Alcoholism is a progressive disease associated with maladaptive changes in behavior that are mediated by environmental and genetic factors, as well as by physiological changes that take place in the brain as a result of exposure to alcohol. Interestingly, genetics and specific environmental factors play an important role in regulating neurogenesis, and these same environmental factors (discussed below) are key factors in the risk of developing alcoholism. Given the overlapping genetic and environmental factors that appear to be involved in both adult neurogenesis and alcoholism, we argue that understanding the commonalities between these two plastic processes may provide new clues to the treatment and prevention of chronic alcoholism.

### Genetic Regulation

Animal genetic studies; classic twin, family, and adoption studies; and systematic searches of the entire human genetic makeup (i.e., the genome) have demonstrated that genetics plays a significant role in the risk of developing alcohol dependence and excessive alcohol consumption (Schuckit 2000). Furthermore, animal studies clearly have indicated that genetic factors influence many responses to alcohol use, including sensitivity to alcohol intoxication, alcohol withdrawal seizures, and preference for drinking alcohol over water (Crabbe 2002).

Likewise, genetics influences the three main components of neurogenesis: NSC proliferation, cell survival, and cell differentiation into neurons and other types of brain cells. For example, in the dentate gyrus of the hippocampus, these components differ for each of the commonly used strains of mice: C57BL/6, BALB/c, CD1, and 129/SvJ (Gage 2000). C57BL/6 mice exhibit the highest rate of NSC proliferation, but the number of cells that actually become neurons (i.e., net neurogenesis) is greatest in the CD1 strain. In contrast, 129/SvJ mice form fewer neurons but form more of the star-shaped brain cells (i.e., astrocytes) that help support neurons’ environment. The formation of fewer neurons results in significantly less hippocampal neurogenesis than in other strains. The volume of the brain cell layer of which these newborn cells eventually become a part (i.e., the granule cell layer of the dentate gyrus) also varies among mouse strains (Gould 1999). Thus, genetic differences make significant contributions to neurogenesis (the new mechanism of brain plasticity), which results in differences in brain structure between animals. The fact that mice share over 80 percent of their genes with humans leads researchers to question whether genetic differences in humans also may underlie differences in brain plasticity or brain structure among individuals. Further studies will need to address the genetic components of adult neurogenesis in models of chronic alcoholism.

### Environmental Regulation

Adult neurogenes also is regulated by environmental factors. Research indicates that animals placed in an enriched environment (in particular, one that promotes physical activity and learning) show a significant increase in neurogenesis compared with animals in normal housing conditions (Gage 2000; Gould 1999). For example, running and hippocampal-dependent learning (such as spatial learning, or learning how to find something in an area) increase NSC survival and differentiation. Furthermore, inhibiting neurogenesis with a drug that prevents cell division disrupts associative learning. Interestingly, this disruption only is observed 1 to 2 weeks after administering the drug, when newborn cells— now functional neurons—would be expected to begin contributing to learning (Gould and Gross 2002). Research comparing different strains of mice indicates that NSCs’ response to environmental stimulation is, at least to some degree, under genetic control. For example, C57BL/6 mice, which have a high innate rate of NSC proliferation, are competent in learning tasks, whereas 129/SvJ mice, which produce fewer neurons than other mouse strains, do not perform well on learning tasks. Thus genetic factors and environmental factors overlap in this new mechanism of brain plasticity.

An important environmental factor is stress. Stress reduces neurogenesis and also is known to precipitate depression and increase drinking. As this environmental factor plays an important role in both neurogenesis and addiction, a more in-depth discussion of this point is included in a later section (see “Alcohol, Stress, and Neurogenesis”).

**Figure:**
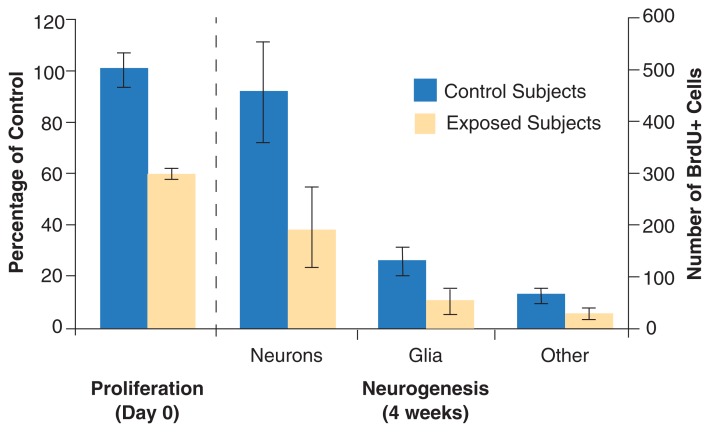
The effect of alcohol on neurogenesis. As shown in the left panel, a single 5g/kg dose of alcohol causes a 40-percent decrease in cell proliferation (newly dividing bromodeoxyuridine [BrdU+]–marked cells) 5 hours later. The right panel shows calculated neurogenesis based on the number of surviving cells 4 weeks after alcohol exposure. By this time, 70 percent of surviving cells (calculated as a percentage of the number of surviving cells in the control group) have migrated and differentiated into neurons, 20 percent have differentiated into glia, and about 10 percent are unclassified. These percentages were multiplied by the number of BrdU+ cells surviving at 4 weeks (right *y* axis), showing the decrease in neurogenesis that resulted from decreased proliferation.

## Alcohol and Dysregulation of Adult Neurogenesis

Although years of research on fetal alcohol syndrome have established that alcohol disrupts the formation of new brain cells in the developing fetus (Crews et al. 2003; Luo and Miller 1998), this article describes the first research into the effect of alcohol on adult neurogenesis.

A single high dose of alcohol reduces NSC proliferation (Nixon and Crews 2002).^3^ One recent experiment in rats showed that a single dose of 5g of alcohol per kg of body weight (a dose that, in humans, would produce blood alcohol levels of about 230 mg/dL, nearly three times the legal driving limit of 0.08 percent) depresses NSC proliferation by 40 percent (Nixon and Crews 2002) (see the figure). As in most recent NSC experiments, the chemical marker bromodeoxyuridine (BrdU) was used to measure cell proliferation (see the sidebar “Measuring Cell Proliferation”). In the adult dentate gyrus, NSCs proliferate in a cell cycle lasting about 25 hours, with the DNA “synthesizing” (or S-phase) lasting about 9.5 hours (Cameron and McKay 2001). The fact that the number of BrdU-positive cells was decreased 5 hours after acute alcohol exposure is consistent with the idea that alcohol inhibits the number of cells entering S-phase, or NSC proliferation. Those cells exiting the 25-hour cell cycle either die or migrate and differentiate into neurons and other brain cells, including glia and oligodendrocytes. As differentiation takes approximately 2 to 4 weeks, any functional effect of reduced proliferation would not be observed until weeks later. Thus, there is a time lag between alcohol consumption and its effects on hippocampal circuitry. As a single dose of alcohol decreases proliferation by 40 percent, this suggests that any observable effects on hippocampal integrity and function would be delayed.

**Measuring Cell Proliferation**

The chemical bromo-deoxyuridine (BrdU) is a thymine analog, meaning that it is similar to thymine (a DNA base) and is incorporated into DNA similarly to thymine. BrdU is incorporated when the cell is dividing and doubling its DNA. Because it can be easily injected into an animal, labels DNA in dividing cells, and can be easily detected by an antibody in tissue sections, BrdU has become the tool of choice for labeling newborn cells. To detect changes in cell proliferation, one merely needs to count the number of labeled cells and compare treatment groups. The accompanying figure shows examples of BrdU-labeled precursors (black cells).

**Figure:**
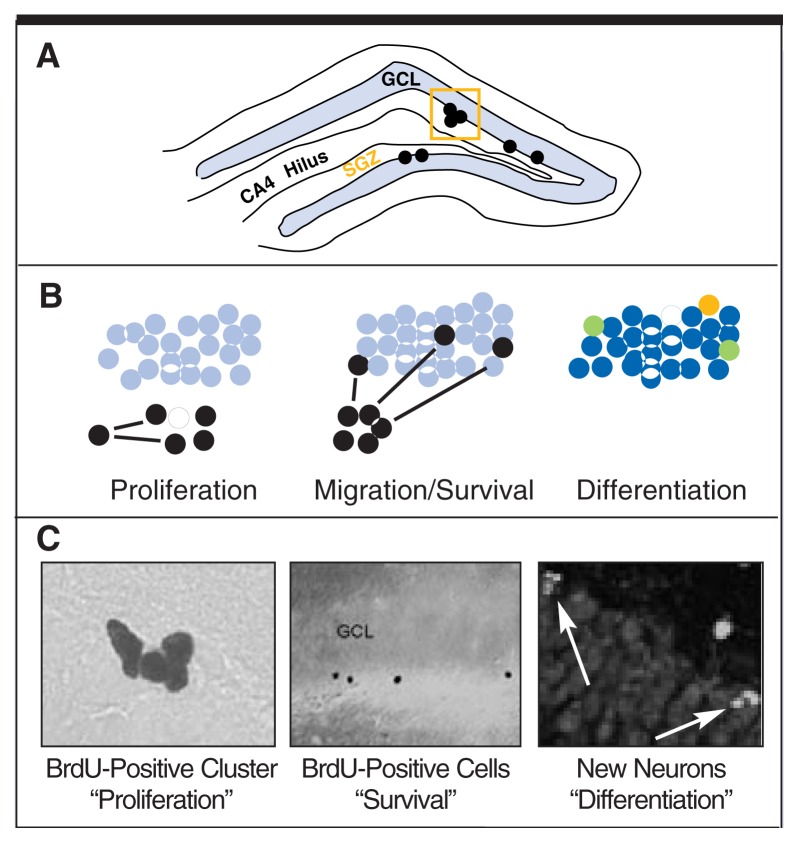
Adult neurogenesis in the dentate gyrus. **(A)** Neural stem cells proliferate along the subgranular zone of the dentate gyrus (shown schematically in the top panel). **(B)** As shown in the cartoon panels, newborn, BrdU-labeled cells (shown in black) proliferate (left), then migrate into the granule cell layer (middle), and eventually differentiate (right) into neurons (blue), glia or oligodendrocytes. As shown in the right-most cartoon panel, cell type, or differentiation, is detected by labeling for multiple proteins/markers in the same tissue. For example, we stain brain tissue sections for cell proliferation (BrdU marker, yellow) and neuron-specific proteins (blue). In the differentiation panel, green cells represent simultaneous labeling of BrdU for newborn cells (in this panel, BrdU is labeled in yellow, rather than black) and for a mature neuron marker (blue). Overlapping blue and yellow make green; these green cells represent recently formed mature neurons in adults. **(C)** Representative photomicrographs (bottom panel) show examples corresponding to each cartoon.

In another experiment in rats, alcohol affected neurogenesis in a 4-day binge-drinking model that produces high blood alcohol levels (equivalent to greater than 300mg/dL in humans, or four times the legal driving limit) and leads to alcohol tolerance and dependence (Nixon and Crews 2002). NSC proliferation was reduced by 53 percent during the intoxication period. In addition, when the survival of those few newly formed cells was investigated 1 month later, few BrdU-labeled cells remained in the alcohol-fed animals, compared with control animals fed the same diet but not given alcohol. These data suggest that alcohol alters the newly formed cells such that normal migration and neuronal differentiation processes may not occur, and the cells do not survive. Recent work has shown that chronic alcohol exposure with lower blood alcohol levels (100–200mg/dL) also affects cell survival (Herrera et al. 2003).

In rats, 6 percent of the total granule cell population is produced each month by NSCs that have divided and differentiated into neurons. Although this figure may seem small, over a lifetime it represents a significant percentage of the neurons. Many years ago, Walker and colleagues (1980) found that feeding animals alcohol for 5 months led to a 20- to 25-percent cell loss in the dentate gyrus, a value close to what would be expected from continuous alcohol-induced inhibition of dentate gyrus neurogenesis. These results suggest that inhibition of the formation of new neurons may contribute to the neurodegeneration associated with chronic alcohol consumption.

A variety of brain insults alter neurogenesis. Lesions (which block cell communication) in key areas of the brain, loss of oxygen (ischemia), or seizures increase NSC proliferation (Duman et al. 2001; Gould 1999). Seizures stimulate NSC proliferation from 3 days to weeks after the seizure. Newly formed cells primarily differentiate into dentate granule cells, many of which appear in abnormal (ectopic) locations and form aberrant connections (Parent et al. 1997). As chronic alcohol exposure can produce seizures, this research raises the question of whether alcohol-induced seizures also increase neurogenesis and aberrant connections.^4^

This discussion has focused on the effect of alcohol use on neurogenesis. Whether neurogenesis or its disruption contributes to alcohol dependence remains to be elucidated by additional research.

### Alcohol, Stress, and Neurogenesis

Stress can be triggered by external events such as the arrival of natural predators, intruders, or a low position in the social hierarchy (subordination hierarchies), or by physiological disturbances such as alcohol consumption. Whatever its origins, stress dramatically depresses NSC proliferation—an effect that persists throughout the chronic stress period and correlates with decreases in the size of the dentate gyrus and with poor learning performance (Gould 1999)—and contributes to the development of alcohol addiction (Kreek and Koob 1998).

Stressors, including alcohol, activate a neuroendocrine network called the hypothalamic–pituitary–adrenal (HPA) axis, which mediates the body’s stress responses (Rivier 1999). Activation of the HPA axis causes the adrenal glands to secrete steroid hormones called glucocorticoids (e.g., corticosterone in rodents), which may underlie the effects of alcohol and other stressors on neurogenesis (Gould 1999). Experimentally administering high levels of glucocorticoids reduces NSC proliferation, whereas reducing glucocorticoid levels in the blood by removing the adrenal glands stimulates NSC proliferation (Gould 1999). However, few if any NSCs have surface proteins (receptors) that bind glucocorticoids, indicating that stress-induced HPA axis activation and the subsequent increase in circulating glucocorticoids must affect NSC proliferation indirectly. This possibility is discussed in more detail below.

## Neurochemical Mechanisms Involved in Alcohol Use and NSC Regulation

### NSC Regulation by Neurotransmitters

Alcohol may affect neurogenesis through its actions on chemicals that bind to neurons and are responsible for nerve signaling (i.e., neurotransmitters). Alcohol is known to alter a variety of neurotransmitter receptors and signals (Crews et al. 1996), and two of these neurotransmitters, glutamate and serotonin, influence adult NSC proliferation.

#### Glutamatergic NSC Regulation

Glucocorticoids—which, as discussed above, are released when alcohol or other stressors activate the HPA axis—are thought to inhibit NSC proliferation by downstream effects on a particular type of glutamate receptor, the *N*-methyl-d-aspartate (NMDA) receptor. Drugs that bind to NMDA receptors and prevent this neurotransmitter’s normal functioning—that is, NMDA receptor antagonists^5^—can interfere with the ability of glucocorticoids to suppress NSC proliferation. This suggests that glucocorticoid levels suppress NSC proliferation indirectly through glutamate neurotransmission or other indirect mechanisms. Although alcohol inhibits NMDA receptors (Crews et al. 1996), this inhibition would be expected to *increase* NSC proliferation, but alcohol clearly *decreases* NSC proliferation in the dentate gyrus (Nixon and Crews 2002). As these findings illustrate, alcohol, glucocorticoids, and glutamate neurotransmission alter adult neurogenesis in complex ways yet to be deciphered.

#### Serotonergic NSC Regulation

A notable correlation exists between conditions that decrease the number of serotonin neurons or nerve terminals in the dentate gyrus and factors that decrease neurogenesis, which suggests that this neurotransmitter is important in the regulation of adult NSCs. Malnutrition, aging, raised glucocorticoid levels, NMDA receptor activation, and depression all are associated with decreases in serotonin fiber density or serotonin release in the dentate gyrus (Gould 1999). Furthermore, inhibiting serotonin synthesis or injuring neurons that release serotonin decreases NSC proliferation in both the SVZ and the dentate gyrus, whereas partially restoring the release of serotonin returns NSC proliferation to normal levels.

Recently, serotonergic regulation of NSC proliferation has been implicated in stress and depression: Experimentally increasing serotonin levels in the hippocampus using antidepressants increases NSC proliferation in the dentate gyrus (Duman et al. 2001; Gould 1999). Indeed, some researchers have suggested that selective serotonin reuptake inhibitors (SSRIs), the most commonly used antidepressants, work by increasing neurogenesis (Duman et al. 2001). Recent studies have found that hippocampal neurogenesis is required for the behavioral effects of antidepressants (Santarelli et al. 2003). Not surprisingly, alcoholism often is associated with mood disorders such as depression and anxiety (Heinz et al. 2001). Thus, the inhibition of neurogenesis by alcohol could contribute to depression secondary to alcoholism. Some studies have found that SSRIs aid in the treatment of depression associated with alcoholism (Naranjo and Knoke 2001), although their effectiveness in treating alcoholism itself has not been well established.

### Neural Stem Cell Regulation by Growth Factors

Like neurotransmitters, proteins called growth factors also bind to neurons and regulate neurogenesis at every stage: proliferation, migration, differentiation, and survival (Cameron et al. 1998). Alcohol may disrupt adult neurogenesis by affecting secretion of growth factors, their targets, or their receptors (Luo and Miller 1998).

Research on fetal alcohol syndrome has contributed to our understanding of the interaction between alcohol and growth factors. In the developing brain, fetal NSCs appear to be highly susceptible to alcohol toxicity, and this is especially true for NSCs that are actively regulated by growth factors (Luo and Miller 1998). Research on fetal alcohol exposure shows that growth factors which cause cells to divide (called mitogenic growth factors)—such as insulin-like growth factor 1 (IGF–1), basic fibroblast growth factor (bFGF), epidermal growth factor (EGF), and platelet-derived growth factor (PDGF)—contribute to the decreased cell proliferation induced by alcohol (Luo and Miller 1998). In fetal cell culture studies, alcohol inhibits growth factor–mediated cell proliferation and survival in several cell types (Luo and Miller 1998). Alcohol also affects IGF–1, a growth factor that increases adult neurogenesis particularly during exercise (e.g., running) (Resnicoff et al. 1994). Thus, growth factors regulate adult neurogenesis and are good candidates for future studies on how alcohol impacts adult neurogenesis.

## Conclusion

Recent findings indicate that NSCs (specifically, in the dentate gyrus of the hippocampus and in the SVC of the anterior lateral ventricles) divide throughout life and give rise to new neurons. This observation has provided a new theoretical framework for understanding processes regulating brain plasticity. The factors that regulate neurogenesis overlap with those that are altered as alcohol use becomes alcoholism (e.g., stress, activity, learning, and other unknown environmental and genetic factors). Although the mechanisms involved are not well understood, it is possible that modulation of neurogenesis contributes significantly to alcoholic pathology.

In animal models, high doses of alcohol have been shown to disrupt neurogenesis, and may underlie long-term deficits in hippocampal structure and function. More moderate but chronic alcohol consumption also affects neurogenesis, suggesting that inhibition of neurogenesis may contribute to the neurodegeneration associated with chronic alcoholism. Alcohol may lead to disruptions in neurogenesis in several ways: through increased levels of glucocorticoids triggered by stress, direct inhibition of glutamate–NMDA receptors, serotonin dysregulation, and inhibition of growth factor–mediated cell proliferation. Research is needed to more directly determine the function of adult neurogenesis and how alcohol-induced inhibition of neurogenesis might contribute both to the pathology and the behavioral changes associated with alcohol abuse.

## Footnotes

1 Vast networks of neurons perform the brain’s essential functions: storing information, regulating basic body functions, and directing behavior. The basis of these networks is communication from cell to cell by chemical messengers called neurotransmitters. Released at narrow gaps, or synapses, between cells, neurotransmitters cross the synapses and activate proteins called receptors. Activated receptors, in turn, induce cascades of molecular interactions within the receiving cell (and, via feedback mechanisms, to the signaling cell as well), resulting in short- and long-term functional changes within the brain.

2 A third question of interest to the alcohol community, though not necessarily addressed in this article: Can stem cells be used to repair brain damage caused by alcohol? The question of brain repair by stem cells is a hot topic; the research discussed here provides the scientific foundation for research into this question. Understanding how alcohol interacts with the resident, or “endogenous,” population of neural stem cells and what happens to these cells in alcoholism is the first step in establishing whether the use of stem cell therapies is an option for treatment (Crews et al. 2003). In addition, great therapeutic potential lies in the pharmacological or behavioral regulation of these endogenous stem cells. Altering endogenous stem cells by behavior or drugs is noninvasive and is a growing area of research.

3 Neurogenesis has been confirmed in the dentate gyrus of the hippocampus of both rodents and humans, thus more research has been conducted on the effects of alcohol on hippocampal neurogenesis than on neurogenesis in the SVZ.

4 Other addictive drugs—including opiates, nicotine, and stimulants—disrupt neurogenesis. Chronic morphine or heroin use inhibits hippocampal neurogenesis similarly to alcohol (Eisch et al. 2000).

5 In contrast to antagonists, agonists are drugs that spur activity at cell receptors which normally are stimulated by naturally occurring substances, triggering a biological response.
